# Efficacy of Phrenic Nerve Catheter in Ipsilateral Shoulder Pain After Thoracic Surgery

**DOI:** 10.7759/cureus.13330

**Published:** 2021-02-13

**Authors:** Linda Le-Wendling, Barys Ihnatsenka, Adrian J Maurer, Yury Zasimovich

**Affiliations:** 1 Anesthesiology, University of Florida College of Medicine, Gainesville, USA

**Keywords:** diaphragm, local anesthetics, phrenic nerve, ropivacaine, shoulder pain, thoracotomy

## Abstract

The mechanism of ipsilateral shoulder pain (ISP) after thoracic surgery remains unexplained definitively in the literature. Regional techniques targeting specific nerves more precisely will provide practitioners with a better understanding of the pain source. We report the case of a 51-year-old woman who underwent robotic-assisted plication of the right hemidiaphragm. ISP was adequately managed using a low-volume infusion through a continuous phrenic nerve block in addition to a thoracic epidural for her chest pain. ISP after thoracic surgery likely originates from diaphragm manipulation. Phrenic nerve blockade is a successful strategy that does not worsen subjective dyspnea in this setting.

## Introduction

Severe ipsilateral shoulder pain (ISP) after thoracic surgery occurs in about 40% to 85% of patients despite successful midthoracic epidural placement and extensive sensory blockade of the chest wall [1-7] and is resistant to opioids, ketamine, and nonsteroidal anti-inflammatory agents and acetaminophen [4,8-10]. Two popular postulated mechanisms of ISP are referred pain due to pleural irritation from the pericardium, mediastinum, and diaphragm via the phrenic nerve or musculoskeletal pain arising from surgical manipulation (e.g., scapular retraction). Understanding the mechanism for shoulder pain would better assist the clinician in selecting the most effective analgesic modality and target location for perineural blockade.

The incidence and intensity of ISP has been reduced with interscalene, supraclavicular, cervical plexus, stellate ganglion, suprascapular blocks more proximally in the supraclavicular fossa (and not distally at the suprascapular notch), and phrenic nerve blocks [2-4,11-15]. The common denominator in these blocks is the likelihood of local anesthetic spread to the phrenic nerve. 

In this case, severe ISP was successfully managed with a low-volume local anesthetic infusion through a continuous phrenic nerve block.

## Case presentation

A 51-year-old woman (body mass index: 22, height: 60 inches) presented to the hospital with a history of anxiety, whose workup of shortness of breath initially led to a diagnosis of asthma though the patient did not improve with inhaled steroids or nebulized bronchodilators. Preoperative pulmonary function test revealed increased expiratory reserve volume, but all other parameters were normal. After further diagnostics including a sniff test under fluoroscopy, she was noted to have right hemidiaphragm elevation and was scheduled to undergo robotic-assisted plication of the right hemidiaphragm via a thoracic approach. We speculated that the patient may experience prolonged shoulder pain. We initially placed a T6/T7 epidural catheter and confirmed the patient had a sensory change to pinprick from T2 to T9 after injection of a test dose consisting of 3 mL of lidocaine 1.5% with 1:200,000 epinephrine. We placed a phrenic nerve catheter (Teleflex, Wayne, PA) using ultrasound guidance (Sonosite Edge II, Sonosite, Bothell, WA) and nerve stimulation (UltraCath Continuous Nerve Block Catheter, Teleflex, Richmond, VA). A hypoechoic non-pulsatile structure was noted arising from the C5 nerve root anteriorly (Video 1).

**Video 1:** Ultrasound scan of the neck The C5 contribution to the phrenic nerve is identified. The scan is performed distal to proximal.

A 17-gauge Tuohy needle was advanced toward this structure located superficially on the anterior scalene muscle belly and rhythmic diaphragmatic contractions were elicited via nerve stimulator. The catheter was threaded 3 to 4 cm past the tip of the Tuohy, maintaining the diaphragmatic motor response at a minimum current threshold of 0.6 mAmp (2 Hz frequency, 0.3 msec pulse wave duration). The catheter was secured with Dermabond (Ethicon Inc, Somerville, NJ), Mastisol (Ferndale Laboratories, Ferndale, MI), and an occlusive dressing (Tegaderm, 3M Medical, St Paul, MN). Injection of a 3 mL test dose (1.5% lidocaine with 1:200,000 epinephrine) ablated this twitch (Videos 2-3). Spread of local anesthetic around the phrenic nerve was noted on ultrasound (Video 4).

**Video 2:** Positive raj test Note the abdominal wall movement from stimulation of the phrenic nerve and the ablation of this twitch with injection of local anesthetic through the phrenic nerve catheter.

**Video 3:** Positive raj test on ultrasound imaging of the diaphragm

**Video 4:** Spread of local anesthetic during injection of a test dose through the phrenic nerve catheter

The thoracic epidural was initiated with an 8 mL/hour basal rate infusion of 0.2% ropivacaine and a 4-mL patient-controlled epidural analgesia bolus every 60 minutes. To determine the presence and severity of ISP in this case and better assess the utility of the phrenic nerve catheter, the catheter was capped and no infusion was initiated. 

Preoperatively, the patient received acetaminophen. Intraoperatively, the patient received 250 mcg of fentanyl, 8 mg of dexamethasone, and 30 mg of ketorolac for the 1.5-hour surgery. Perioperative gabapentin is not routinely used for multimodal analgesia at the author's institution due to increased risk of dizziness and somnolence. Three hours after the initial test dose via the phrenic nerve catheter, the pain service was called to assess severe ISP. The patient denied pain at the surgical site and had good sensory level at her chest. We injected 2 mL of 0.2% ropivacaine through the phrenic nerve catheter and pain subsided to minimal discomfort. An infusion of 0.2% ropivacaine was initiated at 2 mL/hour with good relief of the ISP. The initiation and maintenance of the phrenic nerve catheter did not result in subjective worsening of dyspnea and the patient was noted to be breathing comfortably on assessment by the thoracic surgical team. In addition, sensory and motor testing was performed and demonstrated no motor weakness of the upper extremity (shoulder abduction, elbow flexion) nor sensory changes in the distribution of the brachial plexus.

On postoperative day (POD) 2, as is our routine practice, the regional analgesic pumps were paused to determine the adequacy of pain management with our usual multimodal systemic analgesics prior to removal of the epidural and phrenic nerve catheter. The thoracic epidural was successfully removed on POD 2, but the patient requested that the phrenic nerve catheter infusion be resumed due to her ongoing moderate to severe shoulder pain. The phrenic nerve catheter was successfully removed on POD 4 when the shoulder pain without the nerve block was minimal. 

## Discussion

Severe ISP after thoracic surgery occurs despite successful midthoracic epidural placement. Risk factors are difficult to define. ISP appears to have no relationship to chest tube placement or removal [6,14]. Interestingly, patients with pneumonectomies in which the phrenic nerve had been dissected do not appear to have ISP, lending further credence to the theory that blockade of the phrenic nerve may be important for controlling ISP [7]. In our case, we anticipated a potentially severe ISP due to previous experiences with surgeries of the diaphragm. However, we wanted to confirm the presence of ISP before initiating the phrenic nerve catheter to ensure that 1) ISP was severe enough to warrant initiation of our block; 2) it is the blockade of the phrenic nerve that alleviates this pain and 3) no worsening of respiratory status would be noted with phrenic nerve catheter initiation.

The duration of ISP can be short (~6 hours) or last up to four days, with a median of 23 hours [5]. In this case, surgery was performed on the diaphragm, and the referred shoulder pain from the diaphragm outlasted the somatic chest wall pain from the thoracoscopic port incisions.

Most targeted phrenic nerve blocks are performed by the surgical team by depositing 10 mL of low-concentration local anesthetic in the periphrenic fat pad near the diaphragm [1,3,4,7] However, this technique does not reliably manage ISP (the incidence of ISP remains ~33% with this technique), is short-lived, and cannot be repeated. One study placed non-stimulating nerve catheters in the supraclavicular area either where the phrenic nerve is noted to pull away from C5 or between the sternocleidomastoid and anterior scalene muscles (when the phrenic nerve is not visualized) and 10-mL boluses of local anesthetic vastly improved pain scores for ISP [5]. However, it is hard to separate the blockade of the phrenic nerve from that of the cervical or brachial plexus with this volume of local anesthetic in that area. In this case, successful analgesia with low volumes and rates of low-concentration local anesthetic through a precisely placed phrenic nerve catheter confirmed that our patient’s ISP more likely originated from the phrenic nerve instead of the brachial or cervical plexus. Our patient did not demonstrate upper extremity or shoulder sensory changes or weakness. 

Blockade of the phrenic nerve does not appear to significantly reduce spirometry values or worsen dyspnea in patients undergoing thoracic surgery beyond what is expected for thoracic surgery [3,5]. In our case, the dyspnea perceived by the patient and the patient’s oxygen saturations were not worsened after phrenic nerve blockade. This may be partially because this patient had a diagnosis of hemidiaphragmatic dysfunction with eventration.

Indication for diaphragm surgery may be important in predicting ISP. In the case of a preexisting phrenic nerve injury that results in significant preoperative hemidiaphragmatic paralysis, a phrenic block is unlikely to worsen motor function and may predict a lower likelihood of ISP since pain transmission will also be lost, provided the injury is old and not acute. We observed a previous case in which a patient with preoperative phrenic nerve dysfunction, in whom we were unable to elicit a diaphragm twitch with nerve stimulation preoperatively, did not develop ISP after diaphragm imbrication surgery. 

However, in this case, the patient had eventration, which is elevation of the diaphragm due to redundant nonfunctional diaphragm. We noted right diaphragm movement by visual inspection of the abdominal wall and on ultrasonographic images before phrenic nerve blockade.

A continuous catheter placed preoperatively allows us to have an immediate ability to initiate phrenic nerve blockade and establish its efficacy for ISP. It also allows us to use smaller doses to limit local anesthetic spread to other structures, which allows us to better determine whether the source of the pain can be blocked by targeting the phrenic nerve (and not the brachial or superficial cervical plexus). Furthermore, it allows us to sustain analgesia for multiple days (facilitating assessment of the duration of ISP). We threaded the catheter 3 to 4 cm beyond the needle tip to reduce the potential for catheter migration.

Importantly, our case report differs from previously published literature because we used the patient as her own control for intervention from the standpoint of pain control and respiratory function. We also used low volume, targeted phrenic nerve blockade using a stimulating catheter for more precise catheter tip placement. We deliberately checked upper extremity motor and sensory changes and confirmed that our phrenic nerve catheter spared the cervical and brachial plexus. We believe this case furthers our understanding that blockade of the phrenic nerve (and not the brachial plexus or the superficial cervical plexus) results in effective analgesia for ISP. 

## Conclusions

ISP after thoracic surgery can originate from irritation of the pleura and diaphragm, which is innervated by sensory fibers of the phrenic nerve, and it may be successfully managed with a continuous infusion targeting the ipsilateral phrenic nerve. Phrenic nerve blockade does not appear to worsen subjective dyspnea in this patient population.
